# Parental Education and Adolescents’ Asthma: The Role of Ethnicity

**DOI:** 10.3390/children10020267

**Published:** 2023-01-31

**Authors:** Edward Adinkrah, Babak Najand, Angela Young-Brinn

**Affiliations:** 1 Department of Family Medicine, Charles R. Drew University of Medicine and Science, Los Angeles, CA 90059, USA; 2 Marginalization-Related Diminished Returns Center, Los Angeles, CA 90059, USA

**Keywords:** parental education, risk behavior, asthma, ethnic groups, asthma, adolescents

## Abstract

While high parental education is associated with better health, this association may be weaker for ethnic minority than for ethnic majority families. It is unknown whether the association between parental education and adolescents’ asthma also varies by ethnicity. Aim: To study the association between parental education and adolescents’ asthma overall and by ethnicity. Methods: The current study used data from the Population Assessment of Tobacco and Health (PATH)-Adolescents study. All participants were 12 to 17-year-old non-smokers (n = 8652). The outcome of interest was adolescents’ asthma. The predictor of interest was baseline parental education, the covariates were age, sex, and number of parents present at baseline, and the moderator was ethnicity. Results: According to logistic regression analyses, higher parental education was predictive of adolescents’ asthma; however, this association was weaker for Latino than non-Latino adolescents (OR 1.771; CI 1.282–2.446). We did not find a significant difference in the effect of parental education on asthma of White and African American adolescents. Our stratified models also showed that higher parental education was associated with lower asthma for non-Latino but not for Latino adolescents. Conclusion: The effect of high parental education on adolescents’ asthma prevalence differs between Latino and non-Latino families, with Latino families showing weaker protective effects of parental education on adolescents’ asthma. Future research should test the role of exposure to environmental pollutants, neighborhood quality, and prevalence of smoking in social network members as well as other contextual factors at home, in school, and in the neighborhood that may increase prevalence of asthma in Latino adolescents regardless of their parental education. Given that these potential causes are multi-level, potential causes of such disparities should be tested in future multi-level research.

## 1. Background

Asthma is one of the leading chronic illnesses of adolescents in the United States and worldwide [1]. Asthma is also one of the leading causes of healthcare use by adolescents [2]. On average, in a classroom of 30 children, about three adolescents have asthma [3]. As such, up to ten percent of adolescents may have asthma [4]. Adolescent asthma is one of the main causes of poor quality of life and absenteeism from schools [5]. Given that adolescents asthma is a major public health challenge, we need to study its distribution in the society through epidemiological studies [6,7].

Adolescent asthma is not randomly distributed in communities, and is more common in disadvantaged areas of the society [8]. Adolescents who live in families with higher socioeconomic status (SES) backgrounds are less likely to be diagnosed by asthma [9]. In addition, adolescents with highly educated parents and high-income families may have a lower risk of asthma compared to those with lower education and lower incomes [10]. This is in part because SES protects populations and individuals against some of the causes of asthma, such as allergic responses to infections, stressors, and environmental pollutants and irritants [11].

Some of the mechanisms of the protective effects of SES against youth asthma include house quality, neighborhood quality, school quality, air pollution, and proximity to industrial complexes and freeways [11]. Another mechanism is high SES’s association with the lower tobacco use of peers, parents, family members, and relatives [12]. One of the other effects of high parental education on reducing adolescents’ asthma is the lower risk of exposure to pollutants in high SES environments [13]. For example, secondary tobacco exposure is less common for high SES families [14]. High SES adolescents even have lower cigarette availability in peers, friends, families, and communities [15]. Some other mechanisms are quality of community, neighborhood, school, and home [16].

However, the protective effects of parental education on adolescents’ tobacco use are shown to differ between diverse ethnic groups of adolescents [17]. Marginalization-related diminished returns (MDRs) theory [18] suggests that due to racism and social stratification, family-level resources and assets such as parental education may be associated with lower economic, behavioral, and developmental levels for ethnic minorities than for non-Latino White adolescents [19,20,21]. As a result, disparities such as tobacco use disparities in marginalized and racialized groups (compared to non-Latino White individuals) is sustained across all SES levels [22,23].

A growing body of research has documented ethnic differences in the effects of family SES on adolescents’ health and behaviors that may have implications for asthma [19,20,21,24,25]. The associations between parental education and behaviors that may be correlated with asthma are weaker for ethnic minorities than majority adolescents [26,27,28]. For example, SES’s effects on tobacco use are weaker in ethnic minority than ethnic majority adults and youth [18,19,20,21,24,25,29,30,31,32,33,34,35]. In addition, ethnic minority adolescents attend worse schools than White adolescents across all parental education levels [36]. Similarly, across all parental education levels, ethnic minority adolescents are more likely to have family members who use substances compared to their White peers [37]. These observations are explained via marginalization-related diminished returns (MDRs); family SES resources may generate fewer behavioral, developmental, and health outcomes for marginalized and racialized groups such as African Americans than for non-Latino Whites [22]. While most of the literature that has been produced is on the effects of SES on health outcomes for African Americans [18,19,21,24,25,38,39], SES factors such as parental education may also be associated with lower health gains for other ethnic minorities, such as Latinos and Asians, than for non-Latino White individuals [40]. As this process is similar among all racialized groups, we explain this phenomenon through racism and societal inequalities; even when SES resources are available, societal and environmental conditions such as social stratification, segregation, and racism make it more difficult for ethnic minorities than non-Latino White families to secure outcomes. In this view, what makes a large change for non-Latino Whites may generate smaller real-life changes for ethnic minority individuals [40,41]. However, as most of this is known for African Americans, more research is needed for Latinos.

As high parental education is associated with lower tobacco use in adolescents, it is possible that parental education would also be associated with a lower level of asthma, which would reflect the lower risk of transitioning to tobacco use in the future [42]. However, we are unaware of previous studies that have tested ethnic differences in the association between baseline parental education and subsequent asthma in adolescents.

Built on the MDRs literature on asthma and associated risk factors such as neighborhood quality, stress, environmental quality, and tobacco use [43,44], we conducted this study with two aims; the first was to test the association between parental education and adolescents’ asthma overall. The second aim was to test the variation of this association by ethnicity. Our first hypothesis was that overall, high parental education is associated with a lower prevalence of asthma in adolescents. Our second hypothesis was that this inverse association would be weaker for ethnic minorities than for non-Latino Whites.

## 2. Methods

This was a cross-sectional study with a nationally representative dataset. For this study, we conducted a secondary analysis of the baseline of the Population Assessment of Tobacco and Health (PATH-Adolescents) study data [45]. The PATH-Adolescents is the state-of-the-art study of tobacco use and associated health outcomes such as asthma in US adolescents [46]. Baseline data were collected for wave 1 in 2013–2014 [46]. 

### 2.1. Participants and Sampling

In the PATH study [47], participants are selected randomly. Stratified and clustered random samples were selected from all US states. Eligibility for the PATH sample included non-institutionalized members of US households. All participants were aged between 12 and 17 at baseline. 

### 2.2. Analytical Sample 

For this analysis, we limited the sample to non-Latino and Latino White adolescents who participated in the first wave of the PATH study, regardless of their smoking status. Only non-Latino or Latino White adolescents were enrolled. Overall, the number enrolled totaled 8652.

### 2.3. Study Variables

The study variables in this analysis included ethnicity, parental education, parents’ presence, age, sex/gender, and asthma. Age was a dichotomous variable 0 for lower than 15 and 1 for 15 and above. Gender was 1 for males and 0 for females. Parental education was the independent variable with five levels, and asthma was the outcome. 

#### 2.3.1. Asthma

Asthma was self-reported and measured using the following question: Have you ever been told by a doctor you have asthma? This item was coded as 1 (asthma) to 0 (non-asthma). 

#### 2.3.2. Parental Education

Parental education was a five-level variable, as below: 1 = “no or some high school,” 2 = “Completed high school,” 3 = “Completed college.” This variable was a categorical variable with the lowest education as the reference group.

#### 2.3.3. One- or Two-Parent Household

Family structure was a dichotomous variable that reflected number of parents present in the household.

#### 2.3.4. Ethnicity

Ethnicity was self-identified as non-Latino White or Latino White. Ethnicity was the effect modifier (moderator) and was coded 1 for Latino and 0 for non-Latino.

### 2.4. Data Analysis

Data analysis was performed using SPSS 24. SPSS was used for univariate, bivariate, and multivariable analysis. Univariate was used for descriptive statistics such as mean (standard deviation [SD]) and frequency (%). Bivariate included the Chi-square test. With the outcome being asthma, the predictor variable being parental education, the moderator (effect modifier) being ethnicity, and age, sex, and parents’ presence being the covariates, four linear regression models were applied for multivariable modeling. *Model 1* and *Model 2* were run in the pooled sample. *Model 3* and *Model 4* were performed on non-Latino and Latino adolescents. *Model 1* did not have, and *Model 2* had the interaction term between ethnicity and parental education, i.e., our predictor variable. The odds ratio (OR), SE, 95% CI, and p were reported from each model. We also statistically compared the variance explained by the models across *Model 2* and *Model 1*. Our likelihood ratio test assessed the goodness of fit of *Model 1* and *Model 2,* and showed a more considerable variance of the outcome explained by *Model 2*, with a statistically different model fit. 

### 2.5. Institutional Review Board (IRB)

This study used publicly available PATH data. All data are fully de-identified. Thus, the study was not human subject research and was exempt from a full IRB review.

## 3. Results

8652 adolescents were entered our analysis. Descriptive data are reported in Table 1. 

Table 2 presents the summary of logistic regressions for *Model 1* and *Model 2* that were fitted to the pooled sample. This model shows that higher parental education was associated with lower prevalence of asthma; however, this association was stronger for non-Latino than Latino adolescents.

Table 3 presents the summary of logistic regressions for *Model 3* and *Model 4* that were fitted to non-Latino and Latino adolescents, respectively. As these models show, higher parental education was associated with a lower asthma for non-Latino but not for Latino adolescents.

## 4. Discussion

The current study was performed with two main aims; one to evaluate the overall association between parental education and prevalence of asthma in US adolescents, and the other was to test variation in this association by ethnicity. The first aim showed an inverse association between parental education and prevalence of asthma overall, suggesting that high parental education, as a proxy of SES, is associated with lower prevalence of asthma. The second aim showed moderation of this association by ethnicity. The inverse association between parental education and adolescents’ asthma was weaker for Latino than non-Latino families. 

The inverse association between parental education and prevalence of adolescents’ asthma is in line with theories of fundamental causes, social determinants, social status, status syndrome, and several other models that explain the lower risk of high SES populations and individuals [48,49,50,51,52,53,54,55,56,57,58,59]. According to ecological theories, individuals who live in proximity to low SES neighborhoods, peers, schools, families, and friends will have a higher risk of health problems [60]. Many mechanisms, such as parents and peer risk, quality of neighborhoods, schools, and homes, may explain why high SES is linked to lower prevalence of asthma [61,62,63,64,65,66,67,68,69,70,71]. 

Due to historical racism, social stratification, and segregation, however, ethnic differences exist in the living conditions of high SES individuals across ethnic groups [72,73,74,75]. Multiple studies have documented ethnic minorities’ relative disadvantage in the magnitude of the protective association between SES and health outcomes [18,19,20,21,24,25]. Most of the literature has shown weaker health effects of SES in ethnic minority than ethnic majority adolescents [76]. However, most of these weaker associations between SES and health outcomes are shown for African Americans rather than Latino individuals [18,19,20,21,24,25]. As most past studies are done on African Americans, more research is needed on Latinos. In addition, we are unaware of any past studies on Latino–non-Latino differences in the association between parental education and asthma.

As most past research has been conducted on African American, not Latino, individuals, our observation of a weaker association between parental education and asthma in Latino than non-Latino adolescents is a major advancement of the findings on MDRs. According to marginalization-related diminished returns, resources and assets generate fewer economic, behavioral, developmental, and health outcomes for marginalized groups than for White individuals. 

This study expanded the MDR’s literature, which has, to date, been written on tobacco use [43,44]. Previous work has shown that SES–tobacco use is racialized [43,44]. A study showed that education–tobacco knowledge is also racialized in the US [77]. This finding may be because high-SES White adolescents attend better schools than high-SES ethnic minority adolescents [36]. In addition, there are many challenges in the daily lives of ethnic minority adolescents in US schools [78,79]. Ethnic differences in the returns of education may be because of discrimination in schools [78] or neighborhoods [80]. These MDRs are attributed to social forces rather than biological differences [81,82,83,84,85,86,87,88,89,90,91]. 

### 4.1. Limitations

Our study is not without methodological limitations. First, all variables were self-reported. Thus, our results may be affected by reporting bias and social desirability. Second, our variables were measured at the individual level (reported by adolescents). Some factors may belong to a family or a social network of adolescents. Third, we measured only a few potential confounders, such as gender and age. Other factors, such as the condition of the home, neighborhood SES, and proximity to freeways, were not assessed. In addition, this was a study with an imbalanced sample size (there were a larger number of non-Latino adolescents involved than Latino adolescents). However, our main inference was based on our pooled sample analysis with interaction rather than stratified models. Finally, our study did not evaluate sex differences in the relationship between ethnicity, parental education, and adolescents’ asthma. No causality can established from this study.

### 4.2. Conclusions

To conclude, while overall, high parental education is associated with lower asthma prevalence in adolescents, this inverse association is weaker for Latino than non-Latino adolescents. The diminished returns of parental education on reducing the prevalence of adolescent asthma in Latino families may be due to disproportionate environmental exposures that are related to structural inequalities of racialized families. It is necessary for researchers to explore this observation further.

The family, school, and neighborhood quality of Latino and non-Latino families vary due to the segregation of ethnic minority communities. Additional research is needed to test if ethnic minorities living in ethnic enclaves may show an increased prevalence of asthma across SES levels. Such research would contribute to our understanding of why and how the MDRs contribute to sustained ethnic disparities in asthma for Latino adolescents.

## Figures and Tables

**Table 1:** Descriptive data overall in adolescents (n = 8652).

	Non-Latino n = 6449		Latino n = 2203		All n = 8652	
	n	%	n	%	n	%
Ethnicity						
Non-Latino	6449	0	0	0	6449	74.5
Latino	0	0	2203	100	2203	25.5
Age						
12–14	3212	49.8	1191	54.1	4403	50.9
15–18	3237	50.2	1010	45.8	4247	49.1
Gender						
Female	3108	48.2	1071	48.6	4179	48.3
Male	3330	51.6	1128	51.2	4458	51.5
Two-parent household						
One	1442	22.4	669	30.4	2111	24.4
Two	5005	77.6	1528	69.4	6533	75.5
Parental education						
High School graduated	5785	89.7	1315	59.7	7100	82.1
College graduated	2427	37.6	303	13.8	2730	31.6
Asthma						
No	5340	82.8	1827	82.9	7167	82.8
Yes	1109	17.2	376	17.1	1485	17.2

**Table 2:** Pooled sample models in US adolescents.

	Odds Ratio	Lower Bound	Upper Bound	Sig.
Model 1 (all, main effects)				
Latino	0.966	0.841	1.109	0.624
Male	1.418	1.266	1.589	0.000
Age	1.035	0.925	1.158	0.548
Two-parent household	0.900	0.791	1.025	0.112
Parental education (High School graduated)	1.044	0.889	1.226	0.602
Parental education (College graduated)	0.880	0.773	1.000	0.050
Model 2 (all, M1 + ethnicity interaction)				
Latino	0.625	0.478	0.817	0.001
Male	1.413	1.261	1.583	0.000
Age	1.035	0.925	1.159	0.547
Two-parent household	0.904	0.794	1.029	0.127
Parental education (High School graduated)	0.804	0.653	0.989	0.039
Parental education (College graduated)	0.887	0.770	1.023	0.100
Latino × parental education (High School graduated)	1.771	1.282	2.446	0.001
Latino × parental education (College graduated)	1.071	0.746	1.537	0.710

Outcome: asthma score; data: Population Assessment of Tobacco and Health (PATH).

**Table 3:** Stratified models in non-Latino and Latino adolescents.

	Odds Ratio	Lower Bound	Upper Bound	Sig.
Model 3 (non-latino)				
Male	1.381	1.211	1.574	0.000
Age	1.102	0.967	1.255	0.145
Two-Parent Household	0.920	0.788	1.073	0.289
Parental education (High School graduated)	0.803	0.652	0.989	0.039
Parental education (College graduated)	0.888	0.770	1.024	0.101
Model 4 (Latino)				
Male	1.511	1.203	1.897	0.000
Age	0.860	0.685	1.079	0.192
Two-Parent household	0.864	0.679	1.099	0.235
Parental education (High School graduated)	1.417	1.106	1.815	0.006
Parental education (College graduated)	0.958	0.686	1.337	0.802

Outcome: asthma score; data: Population Assessment of Tobacco and Health (PATH).

## Data Availability

PATH data are available here: https://www.icpsr.umich.edu/web/NAHDAP/series/606 (accessed on 28 January 2023).
